# A study on effect of curcumin on anticerebral aneurysm in the male albino rats

**DOI:** 10.1002/brb3.729

**Published:** 2017-08-01

**Authors:** Li‐Juan Bo, Zhuang Miao, Zhan‐Feng Wang, Kai‐Zhi Zhang, Zheng Gao

**Affiliations:** ^1^ Department of Infectious Disease China‐Japan Union Hospital Ji Lin University Changchun China; ^2^ Department I of Neurosurgery China‐Japan Union Hospital Ji Lin University Changchun China; ^3^ Department of Neurosurgery People's Hospital of Dandong City Dandong China

**Keywords:** a cerebral aneurysm, apoptosis, caspase‐3, curcumin, rats

## Abstract

**Introduction:**

This study investigated the curcumin effect on the cerebral aneurysm. Apoptosis is known to play a fundamental role in the pathogenesis of a cerebral aneurysm. Therefore, we investigated the effect of curcumin on apoptosis of smooth muscle cells of a cerebral aneurysm‐induced male albino rats.

**Methods:**

In this study, the cerebral aneurysm has been induced in the male albino rats by the CaCl_2_ administration. After cerebral aneurysm induction, smooth muscle cells were isolated. Cells were treated with curcumin (25 & 50 mg/kg bwt) for 48 hr.

**Results:**

Curcumin reduced altered mitochondrial morphology significantly, evidenced through fluorescence and confocal study. Curcumin treatment reduced the expression of p53, caspase‐3, and bax/bxl‐2 ratio significantly. Curcumin treatment also reversed the cellular architecture of smooth muscle cell wall significantly. Fluorescence and the confocal study confirmed the reduction in apoptosis in a cerebral aneurysm‐induced smooth muscle cells of male albino rats.

**Conclusion:**

Taking all these data together, it may suggest that the curcumin could significantly reduce the CaCl_2_‐induced cerebral aneurysm through the inhibition of cell apoptosis in the cells.

## 1 INTRODUCTION

Curcumin is a principal curcuminoid of turmeric, which comes under the family of ginger. Turmeric present as desmethoxycurcumin and bis‐desmethoxycurcumin forms. Natural phenols provide the yellow color to turmeric and exist as 1, 3‐diketo and enol forms. The keto form is weaker than enol form (Manolova, Deneva, Antonov, Momekova, & Lambov, 2014). Curcumin has been reported to have antioxidant and anti‐inflammatory effects (Biswas, McClure, Jimenez, Megson, & Rahman, 2005; Dutta, Padhye, Priyadarsini, & Newton, 2005; Lim et al., 2005; Weber, Hunsaker, Abcouwer, Deck, & Vander Jagt, 2005). Thiyagarajan & Sharma (2004) have reported that the immunomodulatory, anti‐inflammatory, antioxidant, and neuroprotective effects. In Chinese traditional medicine, the curcumin has been used to treat mental stress and hypochondriac distensive mania and pain. The development of aneurysms has been reduced following curcumin treatment (Parodi, Mao, Ennis, Pagano, & Thompson, 2006). Curcumin has been reported to act against ischemia reperfusion injury (Fiorillo et al., 2008) and dopaminergic neuronal cell death (Yu et al., 2010). The potential therapeutic effect of curcumin against thoracic and abdominal aortic has been reported (Fan et al., 2012; Hao, Chen, Wang, Dong, & Yang, 2014).

An aneurysm is a condition that often leads to death by rupture and dissection (Jones, Spinale, & Ikonomidis, 2009). A cerebral aneurysm is defined as a cerebrovascular disorder in which weakness in the wall of a cerebral artery and vein causes ballooning of the blood vessel and a localized dilation. Aneurysms in the posterior circulation region have a greater risk of rupture. The basilar artery aneurysms account only 3–5% of total cerebral aneurysms. However, most of the common aneurysms located in the posterior circulation (Brisman, Song, & Newell, 2006). A detailed information and mechanism of cerebral aneurysm formation may provide active therapeutic strategies. There are several studies that have reported that the c‐Jun N‐terminal kinase plays a notable role in the prevention of cerebral artery and abdominal aortic aneurysms (Yoshimura et al., 2005). The c‐Jun N‐terminal kinase signaling pathway involves through apoptosis of vascular walls (Takagi, Ishikawa, Nozaki, Yoshimura, & Hashimoto, 2002). However, curcumin protection against cell apoptosis in a cerebral aneurysm is yet to be investigated. Therefore, this study was aimed to investigate the curcumin on apoptosis in a cerebral aneurysm‐induced male albino rats.

## 2 MATERIALS AND METHODS

### 2.1 Materials

Curcumin (≥80%) and other reagent used in this study were purchased from Sigma‐Aldrich (St. Louis, MO, USA). Mito Tracker Red, Hoechst 33258, and primary antibodies of p53, bax, bcl‐2, and caspase‐3 (Source: mouse) were purchased from Santa Cruz Biotechnology, Inc (Dallas, TX, USA). Smooth muscle cells were isolated according to Leik et al. (Wu, Hofman, & Zlokovic, 2003).

### 2.2 Animals

Male albino rats were obtained from the Shangai Animal House, China (180–200 g). The animal was maintained in well‐cleaned polypropylene cages at standard experimental condition (25 ± 0.5°C, relative humidity 60 ± 5%, and a photoperiod of 12 hr/day). All the animals were treated according to internationally accepted ethical procedures.

### 2.3 Experimental groups

Rats were divided into three groups containing six each.

Group I: Control (an aortic aneurysm)
Group II: Curcumin (10 mg/kg bwt)
Group III: Curcumin (20 mg/kg bwt)

### 2.4 Induction of a cerebral aneurysm

Application of CaCl_2_ has been reported to increase thickening of the cerebral wall in rats. Formation of a cerebral aneurysm was achieved following arterial application of CaCl_2_ in rats. Rats were anesthetized with pentobarbital sodium (40 mg/kg bwt) and connected to a rodent ventilator. Respiratory rate was set at 100 strokes/min in ventilator with tidal volume (3 ml). The cerebral artery was exposed and treated perivascularly with a CaCl_2_ solution (0.5 mol/L) for 20 min with use of presoaked gauze applicator (1.0 × 0.5 × 0.2 cm^3^). Sham‐operated animals were treated with saline for 20 min. After exposure, the gauze applicator was removed, and animals were housed in a temperature‐controlled room under normal 12‐hr light/12‐hr dark laboratory conditions with free access to chow and water (Fan et al., 2012).

### 2.5 Isolation of smooth muscle cells

Smooth muscle cells were isolated from rats with and without cerebral aneurysms. The aneurysm specimen was surgically removed and transferred to PBS with antibiotics (penicillin and streptomycin). Smooth muscle cells were isolated according to Leik and Willey (Wu et al., 2003).

### 2.6 Cell culture

Smooth muscle cells were obtained from rats with and without cerebral aneurysms. Cells were supplemented with DMEM (Dulbecco's Modified Eagle's medium), 10% FBS (Fetal Bovine Serum), and 1% antibiotics (penicillin–streptomycin) for the growth under standard conditions.

### 2.7 Mitochondrial dysfunction

To study apoptosis induction potential of curcumin by disrupting the mitochondrial membrane, we observed the changes in mitochondrial morphology following curcumin treatment. Cells were cultured at a density of 4000 cells/well and grown in the culture medium. Cells were treated with curcumin (25 and 50 mg/kg bwt) for 48 hr. At the end of 48 hr, cells were washed with PBS and exposed to 40 nmol/L Mito Tracker Red for 30 min at 37°C, and then cells were washed and exposed to 5 μg/ml of Hoechst 33258 for 25 min at room temperature. Cells were washed with PBS and viewed under a fluorescent microscope (Axiovert 2000; Carl Zeiss, Germany). Hoechst 33258 staining visualizes the cell nuclei, and Mito Tracker Red stains the mitochondria allowing visualizing alteration in mitochondrial structure due to the curcumin treatment (Moktan & Raucher, 2012).

### 2.8 Fluorescence microscopy

To study apoptosis induction potential of curcumin, we observed the changes in mitochondrial and cytoplasmic morphology following curcumin treatment. Cells were cultured at a density of 4000 cells/well and grown in the culture medium. Cells were treated with curcumin (25 and 50 mg/kg bwt) for 48 hr. At the end of 48 hr, cells were washed with PBS and cells were prepared according to Muthuraman et al. (2016) and viewed under a fluorescent microscope (Axiovert 2000; Carl Zeiss).

### 2.9 Confocal microscopy

To study apoptosis induction potential of curcumin, we observed the changes in mitochondrial and cytoplasmic morphology following curcumin treatment. Cells were cultured at a density of 4000 cells/well and grown in the culture medium. Cells were treated with curcumin (25 and 50 mg/kg bwt) for 48 hr. At the end of 48 hr, cells were washed with PBS and cells were prepared according to Muthuraman et al. (2016) and viewed under a confocal microscope (Olympus, Germany).

### 2.10 ROS measurement

To study ROS‐inducing potential of curcumin, we determined the ROS level after curcumin treatment. Cells were cultured at a density of 4000 cells/well and grown in the culture medium. In our preliminary study (data not shown), cells were treated with different concentrations of curcumin including 5, 10, 15, 20, 25, 30, 35, 40, 45, and 50 mg/kg bwt for 48 hr. We observed a significant effect on ROS induction at doses between 25 and 50 mg/kg bwt of curcumin. Therefore, we selected this range of concentrations for our study. Cells were treated with curcumin (25 and 50 mg/kg bwt) for 48 hr. At the end of 48 hr, cells were washed with PBS and cells were prepared according to Muthuraman et al. (2016) and viewed under a fluorescent microscope (Axiovert 2000; Carl Zeiss).

### 2.11 qPCR

Total RNA was isolated from the control and treated groups. The qPCR was performed using a cDNA equivalent of 10 ng of total RNA. The qPCR experiment was carried out using a cDNA with primers specific for p53, bcl‐2 bax, caspase‐3, and a housekeeping gene GAPDH (Table 1). SYBR Green Master Mix was used according to the manufacturers’ instructions (CFX Connect‐Real‐Time PCR Detection System) (Muthuraman, Ramkumar, & Kim, 2014).

**Table 1:** A list of primers used in this study

Gene	Primers	Sequence
p53	Forward	5′‐TAACAGTTCCTGCATGGGCGGC‐3′
	Reverse	5′‐ AGGACAGGCACAAACACGCACC‐3′
bax	Forward	5′‐TGG AGCTGCAGAGGATGATTG‐3′
	Reverse	5′‐GAAGTTGCCGTCAGAAAACATG‐3′
Caspase‐3	Forward	5′‐TTAATAAAGGTATCCATGGAGAACACT‐3′
	Reverse	5′‐TTAGTGATAAAAATAGAGTTCTTTTGTGAG‐3′
GAPDH	Forward	5′‐GGTCACCAGGGCTGCTTTT‐3′
	Reverse	5′‐ATCTCGCTCCTGGAAGATGGT‐3′

### 2.12 Western blot analysis

Cells were washed with PBS with lysis buffer. The equal amounts of lysate protein samples were investigated SDS‐polyacrylamide gel electrophoresis, and PVDF membrane was used for transferring, and nonspecific binding has been blocked with use of Tris‐buffered saline‐Tween buffer that contained 5% nonfat dry milk. Mouse monoclonal p53, bax, bcl‐2, and caspase‐3 and horseradish peroxidase (HRP) conjugated goat antirabbit IgG for 1 hr. Antibodies were used 1:500 ratio in this study. The protein levels of p53, bax, bcl‐2, and caspase‐3 were determined by using enhanced chemiluminescence method (Muthuraman, Jeongeun, & Eunjung, 2014).

### 2.13 Immunohistochemistry

Immunohistochemical staining experiment was done using the streptavidin–biotin complex method. The primary antibodies were diluted (1:400) and were applied to the prepared sections. Then, sections were incubated at 4°C for overnight. The immune complex was viewed with use of 3, 30‐diaminobenzidine. The slide was counterstained with hematoxylin. The section was dehydrated in alcohols and covered with the use of coverslips (Muthuraman, Senthilkumar, & Srikumar, 2009).

### 2.14 Statistical analysis

All the experimental values were expressed as mean ± SEM. The difference between control and treated groups was compared using Student's *t*‐test. A *p *< .05 was considered statistically significant.

## 3 RESULTS

### 3.1 Effect of curcumin mitochondrial membrane disruption

**Figure 1:**
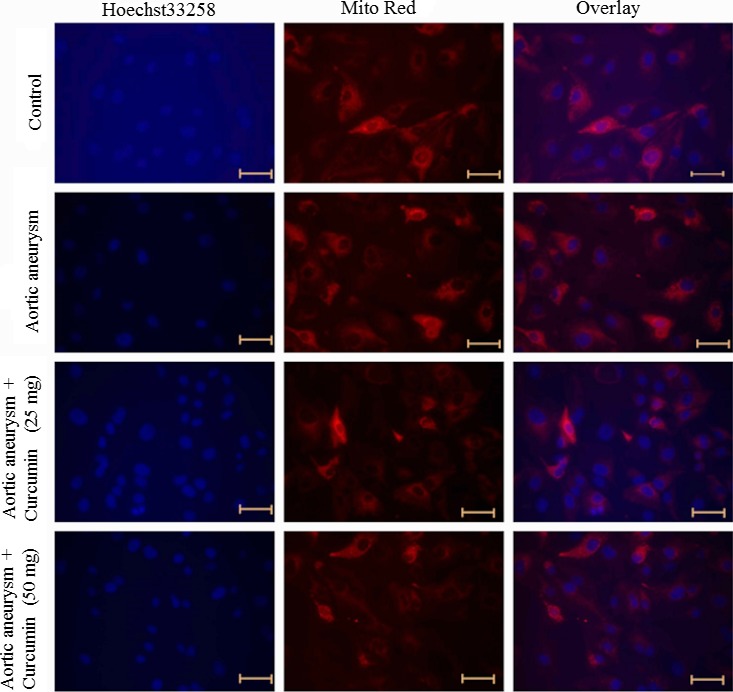
Effect of curcumin on mitochondrial morphology. Cells were exposed to the different concentration of curcumin and stained with Mito Tracker^®^ Red and Hoechst 33258 to image the mitochondria and nuclei, respectively. Scale bar is 50 μm

The changes in the morphology of mitochondria and nucleus in response to the treatment were assessed by the Mito Red and Hoechst 33258, respectively. Normal mitochondria appeared as an extended lace‐like network in control cells, whereas mitochondria were condensed and clumped in the cells of a cerebral aneurysm. Curcumin treatment significantly normalized the mitochondrial morphology toward control (Figure 1).

### 3.2 Effect of curcumin on apoptosis in a cerebral aneurysm

**Figure 2:**
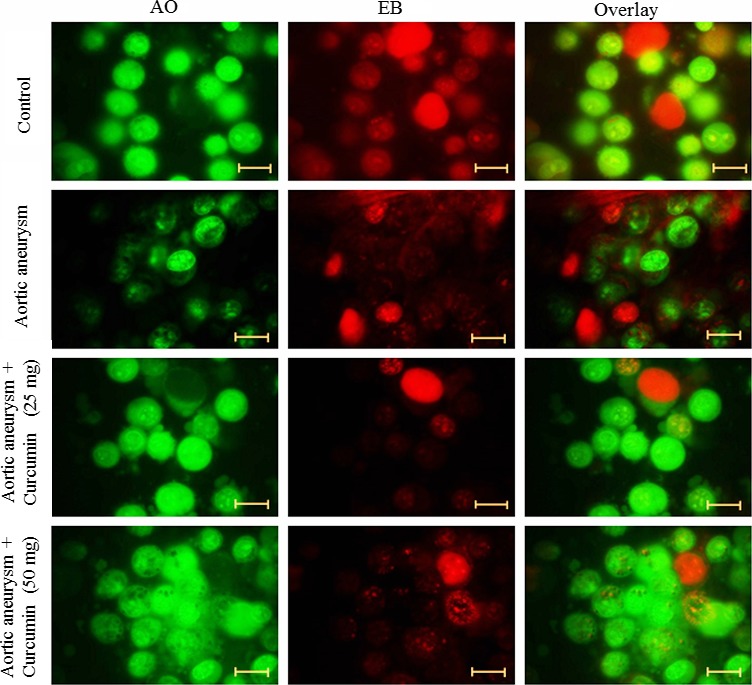
Effect of curcumin on apoptosis. Cells were exposed to the different concentration of curcumin and stained with acridine orange and ethidium bromide. Cells were viewed under a fluorescent microscope. Scale bar is 50 μm

The fluorescence microscopy examination was applied to determine the therapeutic effect of curcumin was involved in the inhibition of apoptosis. Viable cells appear as a green color with a nucleus, and fragmented green chromatin in the nucleus indicated apoptosis. The necrotic cells appear as a uniform bright orange nucleus. Cells incubated with curcumin for 48 hr showed reduced fragmented and condensed chromatin, the appearance of apoptotic cells, and fragmented nuclei (Figure 2).

**Figure 3:**
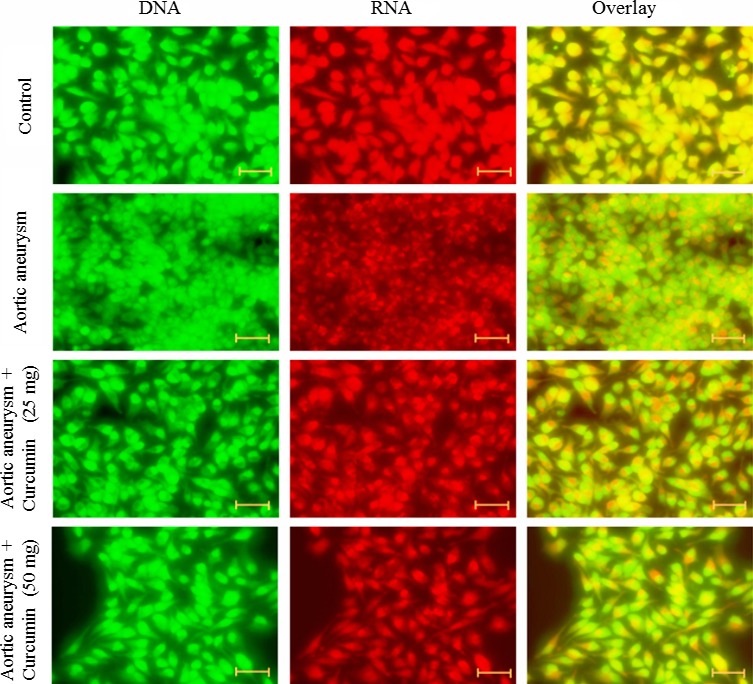
Effect of curcumin on apoptosis. Cells were exposed to the different concentration of curcumin and stained with acridine orange. Cells were viewed under a confocal microscope. Scale bar is 50 μm

Control cells contained smoother and bigger nucleus. Curcumin‐treated cells significantly reduced oval and fragmented granular masses in the nucleus, as well as decreased nuclear volume. The appearance of the nucleus with green color suggesting the induction of apoptosis in curcumin incubated cells (Figure 3).

### 3.3 Effect of curcumin on ROS production of an aortic aneurysm

**Figure 4:**
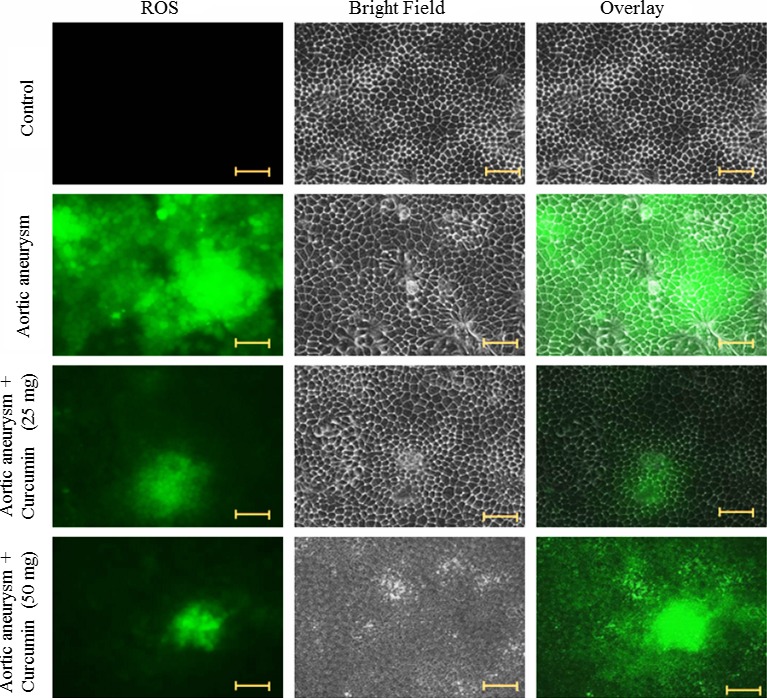
Effect of curcumin on ROS levels. Cells were exposed to the different concentration of curcumin and stained with DCF‐DA. Cells were viewed under a fluorescent microscope. Scale bar is 50 μm

ROS level was determined in the cells incubated with curcumin using fluorescent probe DCFH‐DA dye. DCF fluorescence intensity was decreased in the curcumin‐treated cells in a dose‐dependent relationship manner (Figure 4).

### 3.4 Effect of curcumin on apoptotic‐related gene expressions

**Figure 5:**
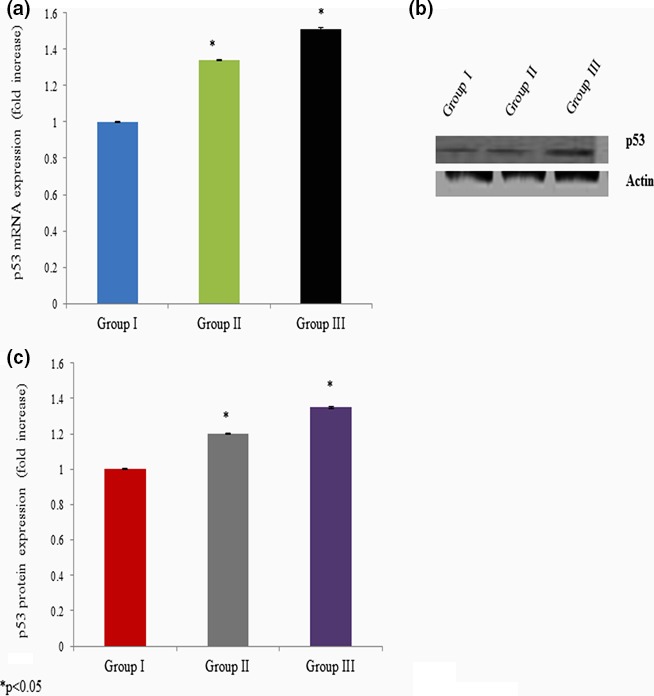
Effect of curcumin on p53 mRNA and protein expression. Cells were exposed to the different concentration of curcumin and qPCR and Western blot analysis. The mRNA expression was expressed as fold change (a), protein expression was given as band (b), and “c” represents the densitometry values of “b”

**Figure 6:**
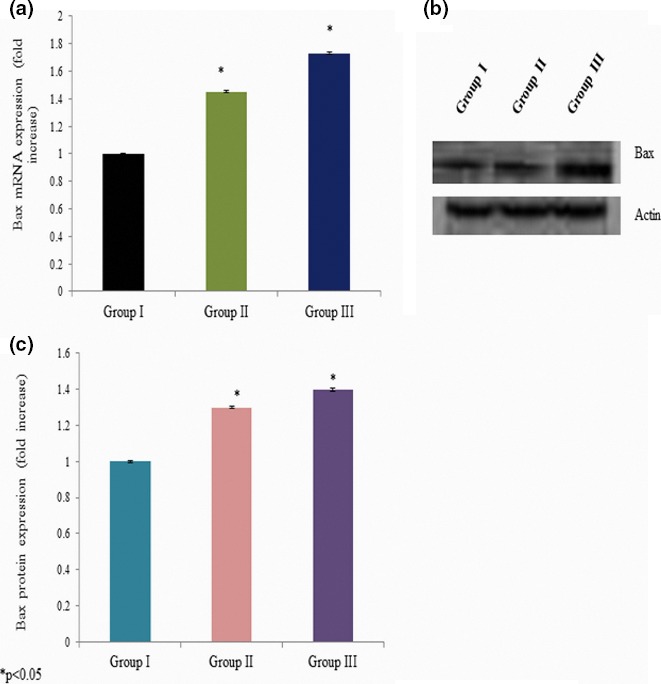
Effect of curcumin on caspase‐3 mRNA and protein expression. Cells were exposed to the different concentration of curcumin and qPCR and Western blot analysis. The mRNA expression was expressed as fold change (a), protein expression was given as band (b), and “c” represents the densitometry values of “b”

**Figure 7:**
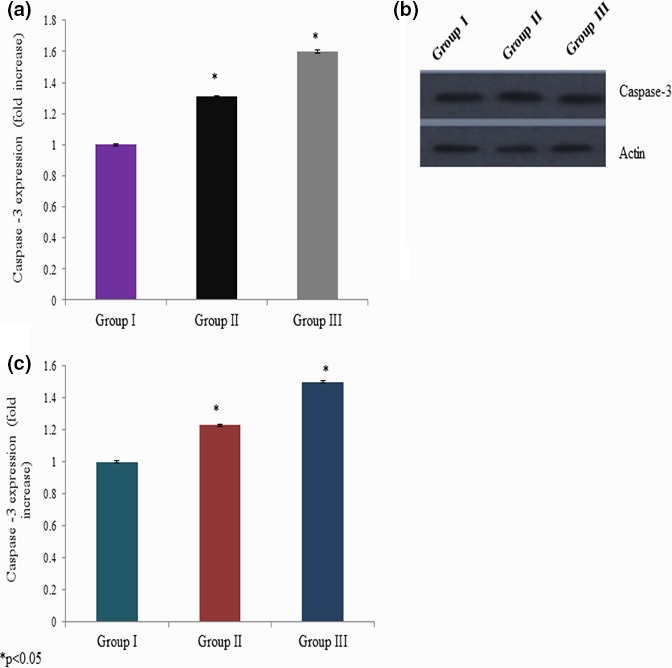
Effect of curcumin on bax mRNA and protein expression. Cells were exposed to the different concentration of curcumin and qPCR and Western blot analysis. The mRNA expression was expressed as fold change (a), protein expression was given as band (b), and “c” represents the densitometry values of “b”

**Figure 8:**
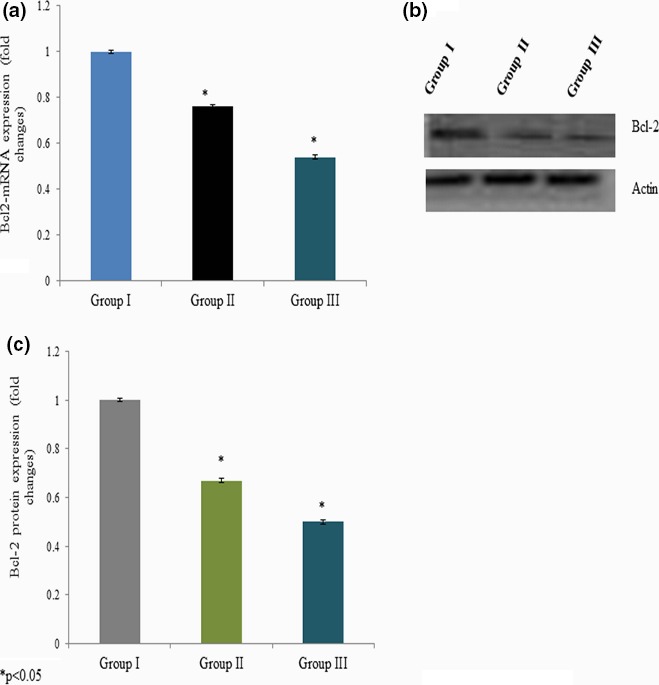
Effect of curcumin on bcl‐2 mRNA and protein expression. Cells were exposed to the different concentration of curcumin and qPCR and Western blot analysis. The mRNA expression was expressed as fold change (a), protein expression was given as band (b), and “c” represents the densitometry values of “b”

We quantitated apoptotic marker gene mRNA expression by qPCR to confirm apoptosis. Cells incubated with different concentrations of curcumin showed significant changes in the p53, bax, bcl‐2, and caspase‐3 mRNA expressions. The p53 mRNA expression was increased 0.34‐ and 0.51‐fold at 25 and 50 mg/kg bwt of curcumin exposure, respectively (Figure 5a). Caspase‐3 mRNA expression was increased 0.45‐ and 0.73‐fold at 25 and 50 mg/kg bwt of curcumin exposure, respectively (Figure 6a). The bax mRNA expression was increased 0.31‐ and 0.60‐fold at 25 and 50 mg/kg bwt of curcumin exposure, respectively (Figure 7a). The mRNA expression of bcl‐2 was significantly reduced 0.24‐ and 0.46‐fold following 25 and 50 mg/kg bwt of curcumin exposure, respectively (Figure 8a).

### 3.5 Effect of curcumin on protein expression

We quantitated apoptotic marker gene expression by Western blot analysis to confirm the effect of curcumin on apoptosis. Cells exposed to various concentrations of curcumin showed significant changes in p53, bax, bcl‐2, and caspase‐3 protein expressions. The p53 protein expression was increased 0.2‐ and 0.35‐fold at 25 and 50 mg/kg bwt of curcumin exposure, respectively (Figure 5b,c). Caspase‐3 protein expression was increased 0.3‐ and 0.4‐fold at 25 and 50 mg/kg bwt of curcumin exposure, respectively (Figure 6b,c). The bax protein expression was increased 0.23‐ and 0.5‐fold at 25 and 50 mg/kg bwt of curcumin exposure, respectively (Figure 7b,c). The protein expression of bcl‐2 was significantly reduced 0.33‐ and 0.5‐fold following 25 and 50 mg/kg bwt of curcumin exposure, respectively (Figure 8b,c).

## 4 DISCUSSION

In the last decades, the anti‐inflammatory and anticancer activity of curcumin has been reported (Wongcharoen & Phrommintikul, 2009), and safety of curcumin consumption and use in clinical treatment has been shown (Chainani‐Wu, 2003). Recently, the curcumin has been reported to reduce aneurysmal degeneration (Parodi et al., 2006). However, the mechanism of curcumin action on cerebral aneurysm yet to be investigated. Therefore, this study examined the key mechanism of curcumin action on cerebral aneurysm using animal cell model. Our experimental data suggest that the curcumin treatment could significantly reduce cerebral degeneration. Curcumin induced damage in the mitochondrial and nuclear genomes in a dose‐dependent manner. However, the mitochondrial damage was greater than nuclear damage. The possible mechanism of curcumin for its effect underlies through the increased ROS and lipid peroxidation. DNA damage was not found at the low level of curcumin treatment. However, it induced DNA damage and oxidative stress at a higher level. Therefore, the nuclear and DNA damage could be the starting point before curcumin‐induced cell death (Jun, Li, Hui‐Min, Yong, & Lai‐Fu, 2006).

The JNK is a mitogen‐activated protein kinase known to play a fundamental role in the development of aneurysms (Yoshimura et al., 2005). The upregulated JNK phosphorylation leads to the destruction of the aorta through the reduction in biosynthetic enzymes and extracellular matrix degeneration. In our experiment, we have used CaCl_2_ to induce a cerebral aneurysm in the male albino rats. The reduction in JNK expression could be helpful to reduce the severity of an aortic aneurysm in the male albino rats. Apoptosis in the cerebral wall has been directly associated with cerebral elastin damage and aortic dilation. The degree of apoptosis in the cells is a crucial mechanism for the prevention and management of an aneurysm (Jones et al., 2009).

The induction of apoptosis has been associated with activation of JNK protein kinase (Xia, Dickens, Raingeaud, Davis, & Greenberg, 1995) and directly associated with phosphorylation of c‐Jun (Force, Pombo, Avruch, Bonventre, & Kyriakis, 1996). Apoptosis in the vessel wall cells has been associated with JNK/c‐Jun pathway in a cerebral aneurysm (Takagi, Ishikawa, Nozaki, Yoshimura, & Hashimoto, 1996). The apoptosis has been reduced in the human smooth muscle cells during the blockage of JNK activation (Han et al., 2010). We studied the effect of curcumin on apoptosis by using fluorescence and confocal microscopy. Apoptosis was significantly reduced in the brain cells after curcumin treatment. Curcumin treatment also reduced JNK expression and reduced the ischemia reperfusion–induced cell death (Fiorillo et al., 2008). Caspase‐3 is an autocatalytic enzyme that plays a key role in apoptosis process. The reduced intracranial aneurysm has been associated with increased caspase‐3 expression (Guo et al., 2007), and our data also agreed on these findings.

The expression of p53, bax, and bcl‐2 had been directly participating in the apoptosis process (Hsu et al., 2011). The ratio of bax/bcl‐2 is very crucial for the determination of cell survival and death (Xu, Liu, Liu, Lu, & Pang, 2011). Curcumin‐induced apoptosis particularly involves the mitochondria‐mediated pathway in different cancer cells. Curcumin induces apoptosis‐like changes in thymocytes, whereas it inhibits cell proliferation in normal cell types (Karunagaran, Rashmi, & Kumar, 2005). Curcumin induces apoptosis through the mitochondria‐mediated apoptotic pathway in HT‐29 cells (Wang, Qi, Zheng, & Wu, 2009). Curcumin‐induced apoptosis by downregulating anti‐apoptotic Bcl‐2 and upregulating proapoptotic Bax, thereby decreasing the Bcl‐2/Bax ratios. Our result about Bcl‐2/Bax ratio is in agreement with previous studies (Shi et al., 2006; Song et al., 2005). Therefore, to confirm the role of curcumin in apoptosis, we further quantitated the expression of p53, bax, and bcl‐2. CaCl_2_‐induced cerebral aneurysm had reduced bax expression and increased bcl‐2 expression. However, curcumin treatment significantly reversed these expression levels in the cells of male albino rats. Taking all these data together, the curcumin treatment significantly protects cells of a cerebral aneurysm.

In other hands, curcumin has been shown to have antioxidant and anti‐inflammatory effects. The chronic inflammation and accelerated oxidative stress could play a key role in prevention and management of a cerebral aneurysm (Ramos‐Mozo et al., 2011). Curcumin treatment has been shown to reduce NF‐kb activation during inflammatory events. Also, curcumin has been reported as a good free radical scavenger by reducing DNA damage and lipid peroxidation (Srivastava, Singh, Dubey, Misra, & Khar, 2011). Curcumin has been reported to induce apoptosis by the activation of caspases through the mitochondrial signaling pathway in melanoma cells (Jiang, Jiang, Li, & Zheng, 2015).

In conclusion, our experimental results show that the curcumin treatment could reduce pathogenesis of CaCl_2_‐induced cerebral aneurysm of brain cells through inhibition of mitochondrial apoptosis process. Furthermore, our experimental result may suggest regular consumption of curcumin could significantly reduce a cerebral aneurysm.

## CONFLICT OF INTEREST

None declared.
